# A framework for assessing the lifetime economic burden of congenital cytomegalovirus in the United States

**DOI:** 10.1186/s12962-019-0189-0

**Published:** 2019-10-03

**Authors:** Aaron Lucas, Anushua Sinha, Karen B. Fowler, Deirdre Mladsi, Christine Barnett, Salome Samant, Laura Gibson

**Affiliations:** 1 RTI Health Solutions, 3040 Cornwallis Road, PO Box 12194, Research Triangle Park, NC 27709-2194 USA; 2 Center for Observational and Real-World Evidence (CORE), Merck & Co., Inc., Kenilworth, NJ USA; 3 University of Alabama at Birmingham, Birmingham, AL USA; 4 University of Massachusetts Medical School, Worcester, MA USA

**Keywords:** cCMV, Framework, Economic, Congenital, Burden of illness, Cytomegalovirus, Cost

## Abstract

**Background:**

In the United States (US), congenital cytomegalovirus infection (cCMVi) is a major cause of permanent disabilities and the most common etiology of non-genetic sensorineural hearing loss. Evaluations of prevention strategies will require estimates of the economic implications of cCMVi. We aimed to develop a conceptual framework to characterize the lifetime economic burden of cCMVi in the US and to use that framework to identify data gaps.

**Methods:**

Direct health care, direct non-health care, indirect, and intangible costs associated with cCMVi were considered. An initial framework was constructed based on a targeted literature review, then validated and refined after consultation with experts. Published costs were identified and used to populate the framework. Data gaps were identified.

**Results:**

The framework was constructed as a chance tree, categorizing clinical event occurrence to form patient profiles associated with distinct economic trajectories. The distribution and magnitude of costs varied by patient life stage, cCMVi diagnosis, severity of impairment, and developmental delays/disabilities. Published studies could not fully populate the framework. The literature best characterized direct health care costs associated with the birth period. Gaps existed for direct non-health care, indirect, and intangible costs, as well as health care costs associated with adult patients and those severely impaired.

**Conclusions:**

Data gaps exist concerning the lifetime economic burden of cCMVi in the US. The conceptual framework provides the basis for a research agenda to address these gaps. Understanding the full lifetime economic burden of cCMVi would inform clinicians, researchers, and policymakers, when assessing the value of cCMVi interventions.

## Background

Approximately 50% of people in the United States (US) have had human cytomegalovirus (CMV) infection by adulthood, as determined by positive serology [1]. The virus is spread by close contact with an infected person through saliva, urine, or other body fluids and also can be transmitted from a pregnant woman to her fetus during pregnancy—termed congenital CMV infection (cCMVi) [2]. In the US, cCMVi occurs in approximately 0.35% to 0.67% of births [3].

About 25% of infants with cCMVi are born with abnormalities such as sensorineural hearing loss (SNHL), chorioretinitis, jaundice, hepatitis, or microcephaly [2, 4]. While most infants are normal at birth, they may develop these abnormalities over time [2, 4]. Regardless of clinical status at birth, approximately 18% of all children with cCMVi develop permanent neurodevelopmental delays or disabilities, often associated with SNHL or vision loss [4]. With cCMVi being the most common congenital infection in the US [5, 6], a significant need exists to reduce its burden.

There are no US Food and Drug Administration (FDA)–approved drug treatments for cCMVi. Off-label use of the antiviral drug ganciclovir and its prodrug valganciclovir are recommended for treating moderate to severe clinical manifestations of cCMVi in the neonatal period, with some clinicians using the antivirals in less severe cases [7]. However, a recent clinical trial demonstrated only modest short-term efficacy for valganciclovir and spurred debate on duration of treatment [8]. Moreover, there are no FDA-approved vaccines or medications to prevent acquisition of CMV during pregnancy or mother-to-fetus transmission, but clinical trials are ongoing [9].

As interventions to prevent the economic consequences of cCMVi are developed, decision makers will require estimates of the economic burden of cCMVi to assess the cost-effectiveness of these new primary and secondary prevention efforts. Conceptual frameworks have been used previously [10, 11] to evaluate the lifetime economic burden of congenital diseases and other conditions and to inform future research agendas (e.g., [10–12]).

With this in mind, the objectives of this study are (1) to develop a conceptual framework to characterize the lifetime economic burden of cCMVi in the US and (2) to use that framework to identify current data gaps for future research into the cost burden of cCMVi in the US.

## Methods

The study’s objectives were addressed in two parts. First, an initial conceptual framework was constructed, then validated and refined in consultation with cCMV experts. Second, for each component of the conceptual framework, costs were either identified from the published literature or flagged as data gaps.

This study focused only on cCMVi-specific costs. Outcomes related to placental CMV infection and inflammation, e.g., fetal loss, preterm delivery, and intrauterine growth restriction, were not included. These important birth outcomes have multiple causes [13, 14], and the fraction attributable to cCMVi is still being defined.

To develop the initial conceptual framework, an impact inventory of costs associated with cCMVi was constructed in accordance with current US methods guidance [15]. Published burden-of-illness studies in congenital diseases [10, 16] were used as models for adapting the impact inventory to cCMVi. The conceptual framework, which was developed by a team of health economists, physicians, and clinical specialists and researchers, included the following types of condition-related costs, per best practices for health economic evaluations [15]:

- Direct health care costs (e.g., tests, physical therapy, occupational therapy).
- Direct non-health care costs (e.g., transportation, social services, education, special housing).
- Indirect costs (e.g., mortality- and morbidity-associated productivity loss for the patient, and caregiver).
- Intangible costs (e.g., increased anxiety or stress or other costs not typically monetized).

In addition, published literature on the classification of impairment, disability, and developmental delay was used to inform development of the framework [17–20]. The World Health Organization (WHO) in The International Classification of Functioning, Disability and Health for Children and Youth (ICF-CY) defines impairment as “a loss or abnormality in body structure or physiological function (including mental functions)” [17; p229] citing examples of paralysis, cardiomyopathy, and deafness. Specific to cCMVi, examples include SNHL, vision loss, and neurological disorders, all of which may vary in severity. According to the WHO’s Early Childhood Development and Disability: A Discussion Paper, impairments may result in developmental delays (“significant variation in the achievement of expected milestones for [an individual’s] actual or adjusted age” [21; p12]) or disabilities (“negative aspects of the interaction between an individual [with a health condition] and that individual’s contextual factors [environmental and personal factors]” [21; p228]). According to the American Academy of Pediatrics [18], delays may occur in one or more areas of physical, language, intellectual, social, or emotional development and may be precursors to disabilities. If a developmental delay or disability is not detected early enough for targeted interventions, it may progress or become permanent [19, 20].

To capture the comprehensive cost of cCMVi, the impact inventory’s costs were examined for when (i.e., at what life stage) and how (e.g., degree of severity of cCMVi-related impairments) these costs would occur. For example, patients with cCMVi may have the bulk of one type of costs occur at one life stage (e.g., antiviral therapy during birth/infancy), while other types of costs may be incurred over a longer time span (e.g., SNHL management throughout childhood and into adulthood). Additionally, patients with severe impairment may use different resources (e.g., supplemental housing, and caregiver time) than patients with mild impairments. Different trajectories for patients with cCMVi were constructed and organized in the form of a chance tree to illustrate the clinical and cost dependencies, and the chance tree was reviewed with experts in the field of cCMV, including two medical epidemiologists (one as a co-author), a physician subspecialist (co-author), a ranking member of a cCMV parent/family advocacy and support organization, a community hospital pharmacy director, and a C-Suite medical director.

To estimate the lifetime cost of cCMVi and identify relevant data gaps in the literature for estimating this cost, an initial targeted literature review was conducted by using keywords to search for US studies in PubMed and Google Scholar (e.g., “congenital CMV” AND [“economic burden” OR “cost” OR “framework”]). Preliminary estimates were discussed with cCMV experts, and the literature was searched again using focused keywords. The literature searches were targeted to fill the trajectories illustrated by the conceptual framework. Tables of relevant and recent cost estimates were compiled, and data gaps were identified.

## Results

### Conceptual framework

Key articles contributing insights to the development of the conceptual framework included cost-effectiveness analyses of interventions or vaccines for cCMVi in the US [22–25]; however, most of these analyses [22–24] relied on data for non-cCMVi-specific patient populations with disabilities that were published more than 25 years ago.

Few published economic studies of cCMVi–specific patient populations were identified. The literature review yielded four studies estimating direct health care costs for patients with cCMVi at birth [26, 27] or in the first year of life [27–29]. Three of the four studies [26–29] relied on the Kids’ Inpatient Database developed for the Healthcare Cost and Utilization Project [30]. The fourth study used two Truven Health Analytics MarketScan Research Databases (IBM Corp., Armonk, New York). No analyses were found that reported the costs of cCMVi after the first year.

**Fig. 1:**
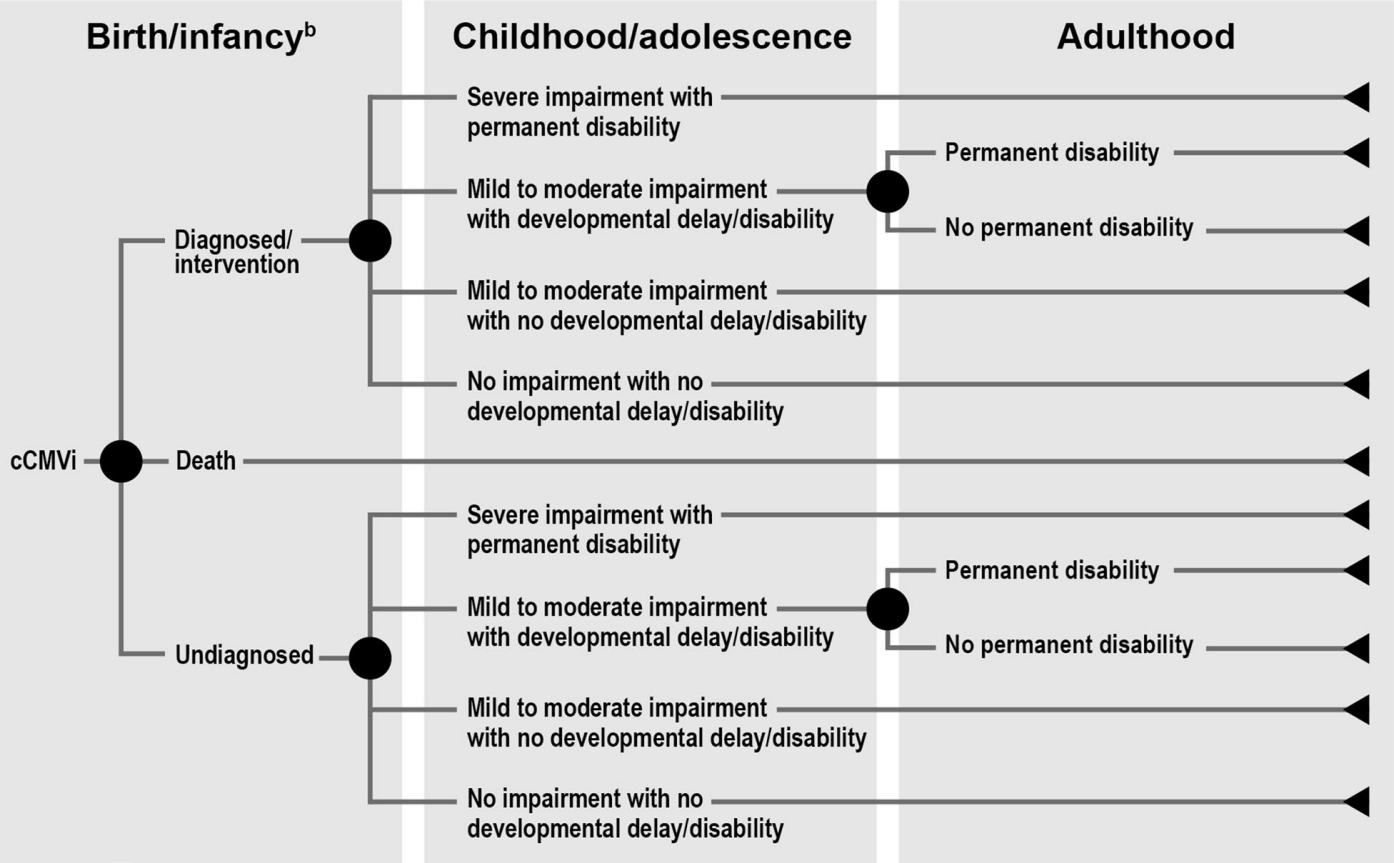
Conceptual framework chance tree for costs of congenital cytomegalovirus infection^a^. cCMVi indicates congenital cytomegalovirus infection. The chance nodes (probabilities) from cCMVi prevention strategies inform the costs of each trajectory. ^a^Identified costs for life stage categories are described in Tables 1, 2, 3. ^b^Birth/infancy costs include costs during first year of life

The conceptual framework (Fig. 1) depicts key cCMVi profiles with economic trajectories corresponding to the life stages of the patient (i.e., birth/infancy, childhood/adolescence, and adulthood). The framework assumes distinct but equivalent trajectories for diagnosed or undiagnosed cCMVi, although the chances of downstream consequences on a trajectory vary depending on diagnosis and any interventions. The framework differentiates trajectories according to the degree of impairment (severe versus mild to moderate) and whether developmental delays or disabilities manifest as a result of impairment. These trajectories were differentiated because they represent substantially different cost profiles over a patient’s life. The framework is agnostic to the types of developmental delays or disabilities included within the categories of mild to moderate and severe impairment. However, for purposes of the literature review, assumptions were made regarding what these categories contain, as described in the results below.

The framework was populated with US-based cost estimates for patients with cCMVi where available. Cost estimates were inflated to 2018 US dollars (US $) using the medical care component of the Consumer Price Index [31].

### Birth/infancy

Table 1 displays estimates of direct health care costs related to cCMVi in the first year of life [26–29]. The studies used to populate Table 1 differed in patient population (all infants with diagnosed with cCMVi [27, 28] vs infants diagnosed with cCMVi and presence of specific symptoms at birth [26, 29]) and type of direct health care cost (all direct health care costs vs. hospitalization costs only [26–29]).

**Table 1:** Birth/infancy, diagnosed/intervention: direct health care costs related to cCMVi at birth/infancy

Cost component	Cost estimate^a,b^	Cost estimate (uncertainty)	Population	Data source	Source
cCMVi-related total direct health care costs per patient					
Birth to < 1 year	Mean: $61,056	SD: $177,404	At least one medical claim with a corresponding diagnosis (i.e., ICD-9-CM code 771.1 or 078.5; ICD-10-CM code P35.1 or B25) and < 1 year of age on the first observed claim for cCMVi	MarketScan CCAE and Medicaid databases	Meyers et al. [27]
Cost per hospitalization					
Birth only	Geo mean: $96,283	SE: $10,507	In-hospital birth with an ICD-9-CM code of 771.1 in any diagnoses listed in the database and symptomatic disease^c^	KID	Inagaki et al. [26]^d^
	Mean, vaginal: $25,203 Mean, caesarian: $32,986	SD, vaginal: $96,443 SD, caesarian: $174,303	ICD-9-CM codes 771.1 or 078.5 or ICD-10-CM code P35.1 or B25 during birth-associated inpatient stay	MarketScan CCAE and Medicaid databases	Meyers et al. [27]^e^
< 1 year (including birth)	Mean, < 1 year of age: $77,538 Mean, < 1 month of age: $93,371	SD, < 1 year of age: $5938 SD, < 1 month of age: $8732	Hospitalization in an infant < 1 year of age with an ICD-9-CM code for cCMVi (771.1), excluding those with codes for HIV (042) or transplant-related diagnoses (transplant [V42], transplant complication [996.8], transplant operation [E87.80]) and a cCMV-related condition^f^	KID	Lopez et al. [29]^g^
	Mean: $107,744	SD: $182,461	A diagnosis of cCMVi (ICD-9-CM code 771.1) and < 1 year of age at admission	KID	Candrilli and Trantham [28]^g^

CCAE, Commercial Claims and Encounters; cCMVi, congenital cytomegalovirus infection; CPI, Consumer Price Index; geo, geometric; HIV, human immunodeficiency virus; ICD-9-CM, *International Classification of Diseases, 9th Revision, Clinical Modification;* ICD-10-CM, *International Classification of Diseases, 10th Revision, Clinical Modification;* KID, Kids’ Inpatient Database; SD, standard deviation; SE, standard error; US, United States

^a^Cost estimates are inflated to 2018 US $ using the medical care component of the CPI [31]. Meyers et al. and Candrilli and Trantham [28] were originally presented in 2016 US $. Lopez et al. [29], and Inagaki et al. [26] were originally presented in 2012 US $

^b^Direct health care cost estimates exclude out-of-pocket costs

^c^Inagaki et al. [26] defined *symptomatic cCMV disease* as “cases with cCMV accompanied by one or more of the following ICD-9-CM codes (codes are diagnosis codes unless specified otherwise): thrombocytopenia (287.3, 287.4, 287.5, 776.1) or requirement for platelet transfusion (procedure code 99.05), petechiae (287.2, 72.6,782.7), hepatomegaly (789.1), splenomegaly (289.51, 789.2), intrauterine growth restriction (764), hepatitis (070.9, 571.4, 571.8, 571.9, 573.1, 573.2, 573.3, 774.4), microcephaly (742.1) or other central nervous system involvement (331.3, 331.4, 742.2, 742.3, 742.4, 779.7), hearing loss (389.1, 389.2, 389.7, 389.8, 389.9, 794.15), or chorioretinitis (363.0, 363.1, 363.20).” [p2]

^d^Inagaki et al. [26] reported the geometric mean of total charges for patients with and without symptomatic cCMV disease. The cCMVi-related cost estimate was calculated as the difference between these two means

^e^Knowledge of a cCMVi diagnosis may affect the choice of delivery. Meyers et al. [27] did not report whether the mother had knowledge of a cCMVi diagnosis before labor. Meyers et al. [27] also reported the difference of mean costs between patients with and without cCMV disease. The population characteristics in the above table were used to define cCMV disease

^f^According to Lopez et al. [29], “Symptomatic cCMV-related conditions were defined using *International Classification of Diseases, 9th Revision*, codes as follows: microcephaly (742.1), hepatomegaly (789.1, 573.1), splenomegaly (789.2), thrombocytopenia (776.1, 287.3, 287.4, 287.5, 776.2), seizures (345, 779.0, 780.39), encephalitis (323, 058), other neurological symptoms (742), cerebral palsy (343), petechiae (772.6, 782.7), hearing loss (389, 315.34, 388.2) and chorioretinitis (363.0, 363.1, 363.2, 363.3).” [p2]

^g^Lopez et al. [29] and Candrilli and Trantham [28] estimated hospitalization costs specifically related to symptomatic cCMVi

Clinical and economic drivers of the costs of cCMVi at birth and through the first year of life were found to be low birth weight [26, 29] and inpatient costs. In addition, inpatient costs were greater during the birth period than any other period during the first year of life [27, 29]. No direct non-health care, indirect, or intangible costs were included in the four studies. Because of this, the total economic burden of cCMVi during the first year of life could not be assessed.

### Death

According to a review of studies of children with cCMVi, the cCMVi-related infant mortality rate for children with symptoms at birth ranges from 5 to 10% in the US [32]. Three of the above studies—Candrilli and Trantham [28], Lopez et al. [29], and Inagaki et al. [26]—found similar mortality rates in their analyses. Estimates of a long-term cCMVi-related mortality rate were not found in the literature. In addition to direct costs and patient (resulting from the loss of a productive lifetime) and caregiver indirect costs, early death of a child due to cCMVi may cause parental loss of productivity or income because of bereavement. Studies that estimate costs associated with premature mortality also may consider costs of living longer, although there are methodological and practical difficulties in doing so [33].

### Severe impairment with permanent disability

The clinical manifestations of severe impairment resulting from cCMVi generally include microcephaly, seizures, and lack of motor function or language [34] and lead to permanent disability. Case reports [35, 36] and interviews with cCMV experts provided detailed descriptions of patients with severe impairment from cCMVi and the medical resources required to support them (Merck Sharp & Dohme Corp, data on file, January 23, 2018). The range of severe impairments described included autism-like syndromes [35], learning disabilities, severe cognitive impairment, and a range of disabilities necessitating special education [36]. Interviews with patient advocates and clinical leaders in the field of cCMV suggest that the lifetime costs of cCMVi may be largest among patients with severe impairment, but these patients may represent a heterogeneous group difficult to study.

Published cost estimates for patients with severe impairment resulting from cCMVi were not available. Congenital conditions, including Zika-associated microcephaly and fetal alcohol spectrum disorder may be similar to the severe impairment resulting from cCMVi [37]. Published studies of these conditions estimate lifetime economic costs in the millions of dollars [38, 39]. The lifetime economic burden of severe impairment resulting from cCMVi may be of the same magnitude.

Lavelle et al. [40] suggested that the economic burden for patients with severe impairment resulting from cCMVi may be weighted heavily toward travel assistance, unpaid family–caregiver time, education support, and supplemental housing. Similarly, intangible costs may include requiring the family to adapt daily schedules to accommodate the combination of impairments. According to the member of a patient/family advocacy and support organization, families of severely affected children may need to move to a different state that provides greater medical benefits aligned with the child’s needs.

### Mild to moderate impairment with no developmental delay/disability

Although cCMVi-related impairments may not interfere with long-term neurodevelopment, managing these abnormalities to maintain normal development may require health care resources. Estimates of the annual direct health care costs related to SNHL and vision loss from any cause range from about $1000 per patient (inflated from 2016 to 2018 US $) [23] for SNHL to almost $8000 per patient (inflated from 2013 to 2018 US $) [41] for vision loss (Table 2).

**Table 2:** Childhood/adolescence and adulthood, mild to moderate impairment with no developmental delay/disability: condition-related annual direct health care costs

Cost component	Cost estimate^a,b^	Cost estimate (variability)	Population	Data source	Source
Direct health care costs related to SNHL					
First year hearing loss is identified	Assumed: $1946 to $2006^c,d^	None reported	N/A	Medicare reimbursement rates applied to treatment algorithms for mild, moderate, and severe SNHL based on clinical opinion	Gantt et al. [23]
< 6 year of age	Assumed: $1921 to $1971^c,d^				
6–12 year of age	Assumed: $1595 to $1682^c,d^				
13–17 year of age	Assumed: $1585 to $1673^c,d^				
≥ 18 years of age	Assumed: $984^c^				
Direct health care costs related to vision loss					
Blindness/vision loss	Total, per person: $7623	None reported	Respondents with ICD-9-CM code 361 (no self-reported diabetes), 362 (no self-reported diabetes), 369, or 378	MEPS	Wittenborn and Rein [41]
Retinal disorders (without diabetes)	Total, per person: $4268				
Strabismus	Total, per person: $2705				

CPI, Consumer Price Index; ICD-9-CM, *International Classification of Diseases, 9th Revision, Clinical Modification;* MEPS, Medical Expenditure Panel Survey; N/A, not applicable; SNHL, sensorineural hearing loss; US, United States

^a^Cost estimates are inflated to 2018 US $ using the medical care component of the CPI [31]. Gantt et al. [23] and Wittenborn and Rein [41] were originally presented in 2016 and 2013 US $, respectively

^b^Direct health care cost estimates exclude out-of-pocket costs

^c^Gantt et al. [23] does not report the type/measure of the cost estimate provided. Cost estimate from Wittenborn and Rein [41] does not control for the presence of other vision-related conditions

^d^The lower bound provides the cost estimate for patients with mild to moderate hearing loss; the upper bound proves the cost estimate for patients with severe to profound hearing loss

Health care resource use for SNHL may include hearing device components, e.g., cochlear implants, which require surgery. Health care costs may be compounded over time (e.g., cochlear implants typically need to be replaced as the patient ages) [42]. Direct non-health care costs (e.g., educational support for missed classes) and indirect costs (e.g., lost productivity for missed workdays) may be incurred [23]. Moreover, intangible costs related to undergoing surgery, including heightened anxiety and short-term decreases in quality of life, also may arise.

### Mild to moderate impairment with developmental delay/disability in childhood/adolescence

Although cost estimates for developmental delays and disabilities were not available in the literature for patients with cCMVi specifically, recent estimates for proxy conditions, e.g., autism spectrum disorder (ASD), attention-deficit/hyperactivity disorder, and cerebral palsy (CP), were available. Table 3 presents the annual condition-related direct health care and non-health care costs for patients in childhood/adolescence with these proxy conditions. For patients in childhood/adolescence with ASD, annual condition-related direct health care and non-health care costs were roughly $4000 and $17,000 per patient, respectively (inflated from 2011 to 2018 US $) [40]. For patients in childhood/adolescence with CP with or without intellectual disability (ID), annual condition-related direct health care costs ranged from roughly $22,000 (CP without ID) to $62,000 (CP with co-occurring ID) (inflated from 2005 to 2018 US $) [43].

**Table 3:** Childhood/adolescence and adulthood, mild to moderate impairment with developmental delay/disability: condition-related annual direct health care and non-health care costs

Cost component	Cost estimate^a,b,c^	Cost estimate (variability)	Population	Data source	Source
Direct health care costs related to mild to moderate impairment with developmental delay/disability in childhood/adolescence					
Out of pocket	Total, per person, ASD: $219	CI, ASD: −$7 to $359	Children 3–17 years of age with ASD^b^	MEPS linked to the NHIS	Lavelle et al. [40]
Third-party payers	Total, per person, ASD: $3629	CI, ASD: $1222 to $5118	Children 3–17 years of age with ASD^b^	MEPS linked to the NHIS	Lavelle et al. [40]
	Total, per person, ID without CP: $22,788 Total, per person, CP without ID: $22,333 Total, per person, CP with ID: $61,837	Not calculated^d^	Children < 19 y of age with CP using ICD-9-CM code 343.xx with at least one inpatient or two outpatient visits that were > 29 days apart during the study period Patients with a medically diagnosed ID (identified as one inpatient or one outpatient claim with an ICD-9-CM code of 317.xx-319.xx)	MarketScan Medicaid Multi-State Database	Kancherla et al. [43]
Direct non-health care costs related to mild to moderate impairment with developmental delay/disability in childhood/adolescence					
Education costs	Total, per person, ASD: $10,346	CI, ASD: $7925 to $12,522	Nationally representative panel of caregivers and parents of children 3–17 years of age who had a child diagnosed with ASD^e^	Administered survey	Lavelle et al. [40]
Condition-related therapy and family-coordinated services	Total, per person, ASD: $421	CI, ASD: −$91 to $1168	Nationally representative panel of caregivers and parents of children 3–17 years of age who had a child diagnosed with ASD^e^	Administered survey	Lavelle et al. [40]
Unpaid caregiver time costs	Total, per person, ASD: $6115	CI, ASD: −$2009 to $14,342	Nationally representative panel of caregivers and parents of children 3–17 years of age who had a child diagnosed with ASD^e^	Administered survey	Lavelle et al. [40]
Direct health care costs related to permanent disability in adulthood					
Out of pocket	Mean: $1477	SD: $2631	Adults 21–61 years of age with self-reported functional and cognitive limitations	MEPS	Mitra et al. [47]
Third-party payers	Mean: $12,773	SD: $38,874	Adults 21–61 years of age with self-reported functional and cognitive limitations	MEPS	Mitra et al. [47]

ASD, autism spectrum disorder; CP, cerebral palsy; CPI, Consumer Price Index; ICD-9-CM, *International Classification of Diseases, 9th Revision, Clinical Modification;* ID, intellectual disability; MEPS, Medical Expenditure Panel Survey; NHIS, National Health Interview Survey; SD, standard deviation; US, United States

^a^Cost estimates are inflated to 2018 US dollars using the medical care component of the CPI [31]. Lavelle et al. [40], Kancherla et al. [43], and Mitra et al. [47] were originally presented in 2011, 2005, and 2004 US dollars, respectively

^b^Lavelle et al. [40] used the publicly available MEPS (2003-2008) linked to the NHIS (2001–2007) to estimate annual total health care expenditures incurred by children aged 3–17 years who responded affirmatively to the question, “Has a doctor or health care provider ever told you that [child’s name] has autism?” and by those in a corresponding control group. Health care utilization counted by the expenditures included all categories reported in MEPS, but out-of-pocket costs were taken from household payments only

^c^Condition-related cost estimates from Kancherla et al. [43] were calculated by taking the difference in costs for patients with the condition versus patients in the control group. Condition-related cost estimates from Mitra et al. [47] were calculated by taking the difference in costs for disabled patients versus non-disabled patients. Condition-related cost estimates from Lavelle et al. [40] were taken from the reported regression-adjusted difference in costs for children with ASD compared with those for children without ASD

^d^Kancherla et al. [43] presented confidence intervals for the mean costs, but not for the condition-related differences reported here

^e^Lavelle et al. [40] used two surveys in 2011 to estimate non-health care expenditures from a nationally representative panel of caregivers and parents of children aged 3–17 years who have a child diagnosed with ASD and a corresponding control group. Reported tuition expenditures from all sources related to school were used to estimate education costs. Condition-related therapy and family-coordinated services include “treatments such as applied behavioral analysis, sensory integration, and communication therapies.” All other resources used to care for children are categorized as “family-coordinated services.” Unpaid caregiver time was estimated as the total time all caregivers in the child’s household spent on “activities such as coordinating their child’s therapies, homework help, and travel to appointments and activities during the previous 12 months.” Some out-of-pocket estimates for direct non-health care cost categories were reported as negative

The economic costs associated with developmental delays and disabilities may include managing significant learning, behavioral, or motor dysfunction [37, 44, 45]. Moreover, these patients may need special education, caregiver attention, or supplemental housing in addition to routine medical care [40, 46]. Interviews with cCMV experts suggested that parents of children with cCMVi exhibiting even mild developmental delays or disabilities may experience costs associated with increased anxiety, depression, or familial tension (Merck Sharp & Dohme Corp, data on file, January 23, 2018).

### Permanent disability

Patients with cCMVi who experience developmental delays or disabilities in childhood/adolescence may progress into adulthood with permanent disabilities. Cost estimates of adults with disabilities resulting from cCMVi specifically were not found in the literature, but one study [47] of adult, non-elderly patients with disabilities reported an annual incremental direct health care cost of roughly $14,000 per year compared with similar patients without disabilities (Table 3, inflated from 2004 to 2018 US $).

## Discussion

Assessing the full economic burden of cCMVi is essential for estimating the economic value of primary and secondary interventions, e.g., screening, vaccination, and clinical management to prevent impairment and permanent disabilities. Prior to this research, no comprehensive assessment of the lifetime economic burden of cCMVi had been presented in the published literature. This study developed a conceptual framework following current US guidelines for health economic evaluations [15]. The conceptual framework was developed by a team of economists and clinical experts in cCMV and informed by published literature and guidance from stakeholders in the patient advocacy, hospital, health insurance, and public health fields. The framework was used to direct searches for published cost estimates for cCMVi and to identify key data gaps in populating the framework, of which many were identified (see Additional file 1: Table S1). The findings from this qualitative project provide the basis for a research agenda to address these gaps.

Although the conceptual framework identified an array of cost impacts relevant to the lifetime economic burden of cCMVi, research published to date has focused primarily on direct health care costs during the first year of life. No published studies of direct non-health care, indirect, and intangible costs were identified. No published work was found that addressed costs of severe impairment resulting from cCMVi or costs for adult patients.

Some research suggests that cCMVi may result in conditions similar to ASD and CP [35, 46], but no studies of the costs of these disorders in patients with cCMVi were identified. Authors of previous economic analyses for cCMVi interventions have considered the economic consequences of cCMVi but without having cCMVi-specific costs to populate their models (e.g., [16, 22, 24] [non-US]). In two of these analyses [24] and Lawrence et al. [22] cost estimates for ID, vision loss, and SNHL were added together in different combinations to reflect the heterogeneity of cCMVi outcomes. However, the total economic burden of patients with cCMVi may be compounded by each individual consequence of the infection [43].

To address data gaps pertaining to the lifetime economic burden of cCMVi in the US, researchers have several potential resources, including health insurance claims databases (e.g., those maintained by IBM), publicly available health surveys, disease registries (e.g., the National Congenital CMV Disease Registry), and parent/family advocacy and support organizations (e.g., The National CMV Foundation). However, these data sources have limitations with regard to informing lifetime cost studies. For example, health insurance claims databases have a relatively restricted period of follow-up because of movement across plans (e.g., [48] suggest that 20% of commercially insured people in the US change insurers annually). Therefore, distinguishing congenital and postnatal CMV infection may not be feasible in a health insurance claims database.

Disease registries have a period of follow-up that is likely to be much longer than a typical claims database. Registries also may offer data indicating direct and indirect costs, although the data may not be collected systematically or be complete given that these registries’ missions may not refer explicitly to assessing the economic burden of cCMVi (e.g., as in the National Congenital CMV Disease Registry) [49].

Additional research using existing data sources may be valuable, but for cCMVi such sources may have limitations that could make primary data collection studies or hybrid studies (i.e., complementing existing data sources with primary data collection) necessary to fill the gaps. For example, an inventory of cCMVi patient registries and the data they collect as well as their capacity to link to other data sources (e.g., US health insurance claims data) or expand the data collected (e.g., via a survey of registry participants or their families) would be informative to potential researchers. Linking data sources also may help researchers characterize the costs incurred before patients are diagnosed with cCMVi or associated with a delayed cCMVi diagnosis.

The methods used for this study were similar to those used in other disease areas (e.g., [10, 12]) and relied in part on targeted reviews of the published literature. Conducting a systematic literature review to develop the conceptual framework would have introduced premature and potentially artificial restrictions. Instead, a more pragmatic, targeted literature review, customized to identify the studies most likely to reveal the potential lifetime cost of cCMVi was appropriate. The targeted literature reviews identified costs and data gaps in the existing published data characterizing the economic burden of cCMVi. The costs presented here may be useful for researchers quantifying the economic burden of cCMVi. However, because of the differences in study designs, caution should be taken when evaluating the costs of cCMVi presented in the literature.

The conceptual framework and resulting analysis presented here were developed using the current state of pertinent knowledge of cCMVi. The field of cCMVi research is growing, so the assumptions underlying the conceptual framework may evolve as researchers make new discoveries. As new primary and secondary interventions for cCMVi are developed, decision makers must assess the implications for cCMVi management guidelines. To evaluate accurately the cost-effectiveness and affordability of new interventions, comprehensive estimates of the lifetime economic burden of cCMVi will be required.

## Conclusions

This study has highlighted significant data gaps, particularly with respect to estimates of direct health and non-health care costs for adult patients and those with severe impairment, costs incurred prior to an established cCMVi diagnosis, and indirect and intangible costs. Existing data sources, e.g., health insurance claims databases and registries, may provide valuable data, but new primary data collection studies are necessary to capture the range of costs associated with cCMVi. The conceptual framework developed in this study may serve as a helpful foundation for future research in estimating these costs.

## Supplementary information

**Additional file 1: Table S1.** Gaps in cost components for estimating the economic burden of cCMVi.

## Data Availability

Not applicable.

## Abbreviations

ASD: autism spectrum disorder
CCAE: Commercial Claims and Encounters
cCMV: congenital CMV
cCMVi: congenital CMV infection
CMV: cytomegalovirus
CP: cerebral palsy
CPI: Consumer Price Index
FDA: US Food and Drug Administration
HIV: human immunodeficiency virus
ICD-9-CM: *International Classification of Diseases, 9th Revision, Clinical Modification*
ICD-10-CM: *International Classification of Diseases, 10th Revision, Clinical Modification*
ICF-CY: International Classification of Functioning, Disability and Health for Children and Youth
ID: intellectual disability
KID: Kids’ Inpatient Database
MEPS: Medical Expenditure Panel Survey
N/A: not applicable
NHIS: National Health Interview Survey
SD: standard deviation
SE: standard error
SNHL: sensorineural hearing loss
US: United States
WHO: World Health Organization

## References

[1] Bate SL, Dollard SC, Cannon MJ (2010). Cytomegalovirus seroprevalence in the United States: the national health and nutrition examination surveys, 1988–2004. Clin Infect Dis.

[2] Centers for Disease Control and Prevention (CDC). Cytomegalovirus (CMV) and congenital CMV infection. 2018. http://www.cdc.gov/cmv/index.html. Accessed 29 Oct 2018.

[3] Kenneson A, Cannon MJ (2007). Review and meta-analysis of the epidemiology of congenital cytomegalovirus (CMV) infection. Rev Med Viro..

[4] Manicklal S, Emery VC, Lazzarotto T, Boppana SB, Gupta RK (2013). The “silent” global burden of congenital cytomegalovirus. Clin Microbiol Rev.

[5] Thackeray R, Wright A, Chipman K (2014). Congenital cytomegalovirus reference material: a content analysis of coverage and accuracy. Matern Child Health J.

[6] Bristow BN, O’Keefe KA, Shafir SC, Sorvillo FJ (2011). Congenital cytomegalovirus mortality in the United States, 1990–2006. PLoS Negl Trop Dis..

[7] Rawlinson WD, Boppana SB, Fowler KB, Kimberlin DW, Lazzarotto T, Alain S (2017). Congenital cytomegalovirus infection in pregnancy and the neonate: consensus recommendations for prevention, diagnosis, and therapy. Lancet Infect Dis..

[8] Kimberlin DW, Jester PM, Sánchez PJ, Ahmed A, Arav-Boger R, Michaels MG (2015). National Institute of Allergy and Infectious Diseases Collaborative Antiviral Study Group. Valganciclovir for symptomatic congenital cytomegalovirus disease. N Engl J Med..

[9] ClinicalTrials.gov. Search results for “congenital CMV”. https://clinicaltrials.gov/ct2/results?term=congenital+CMV&Search=Search. Accessed 29 Oct 2018.

[10] Popova S, Stade B, Lange S, Rehm J (2012). A model for estimating the economic impact of fetal alcohol spectrum disorder. J Popul Ther Clin Pharmacol..

[11] Ericson L, Magnusson L, Hovstadius B (2017). Societal costs of fetal alcohol syndrome in Sweden. Eur J Health Econ..

[12] Barnett CL, Mladsi D, Vredenburg M, Aggarwal K (2018). Cost estimate of platelet transfusion in the United States for patients with chronic liver disease and associated thrombocytopenia undergoing elective procedures. J Med Econ..

[13] Goldenberg RL, Goepfert AR, Ramsey PS (2005). Biochemical markers for the prediction of preterm birth. Am J Obstet Gynecol.

[14] Resnick D, Manolagas S, Niwayama G, Fallon MD (2002). Histogenesis, anatomy and physiology of bone. Diagnosis of bone and joint disorders.

[15] Sanders GD, Neumann PJ, Basu A, Brock DW, Feeny D, Krahn M (2016). Recommendations for conduct, methodological practices, and reporting of cost-effectiveness analyses: second panel on cost-effectiveness in health and medicine. JAMA.

[16] Halwachs-Baumann G (2011). Congenital cytomegalovirus infection: epidemiology, diagnosis, therapy.

[17] World Health Organization. International classification of functioning, disability, and health: children & youth version: ICF-CY. 2007. http://apps.who.int/iris/bitstream/handle/10665/43737/9789241547321_eng.pdf;jsessionid=22327C651B903F692CC909D5CAE57969?sequence=1. Accessed 29 Oct 2018.

[18] American Academy of Pediatrics (AAP). Ages & stages. 2018. https://www.healthychildren.org/english/ages-stages/pages/default.aspx. Accessed 29 Oct 2018.

[19] American Academy of Pediatrics (AAP). Language delays in toddlers: information for parents. 2011. https://www.healthychildren.org/English/ages-stages/toddler/Pages/Language-Delay.aspx. Accessed 29 Oct 2018.

[20] Cheng ER, Palta M, Kotelchuck M, Poehlmann J, Witt WP (2014). Cognitive delay and behavior problems prior to school age. Pediatrics.

[21] World Health Organization (WHO). Early childhood development and disability: a discussion paper. 2012. http://apps.who.int/iris/bitstream/handle/10665/75355/?sequence=1. Accessed 29 Oct 2018.

[22] Lawrence RS, Durch JS, Stratton KR (2000). Vaccines for the 21st century: a tool for decisionmaking.

[23] Gantt S, Dionne F, Kozak FK, Goshen O, Goldfarb DM, Park AH (2016). Cost-effectiveness of universal and targeted newborn screening for congenital cytomegalovirus infection. JAMA Pediatr..

[24] Dempsey AF, Pangborn HM, Prosser LA (2012). Cost-effectiveness of routine vaccination of adolescent females against cytomegalovirus. Vaccine..

[25] Williams EJ, Gray J, Luck S, Atkinson C, Embleton ND, Kadambari S (2015). First estimates of the potential cost and cost saving of protecting childhood hearing from damage caused by congenital CMV infection. Arch Dis Child Fetal Neonatal Ed.

[26] Inagaki K, Blackshear C, Palmer A, Hobbs CV (2018). Risk factors, geographic distribution, and healthcare burden of symptomatic congenital cytomegalovirus infection in the United States: analysis of a nationally representative database, 2000–2012. J Pediatr.

[27] Meyers J, Sinha A, Samant S, Candrilli S (2019). The economic burden of congenital cytomegalovirus disease in the first year of life: a retrospective analysis of health insurance claims data in the United States. Clin Ther.

[28] Candrilli SD, Trantham L (2017). The economic burden of congenital cytomegalovirus-related hospitalizations in the United States. Value Health..

[29] Lopez AS, Ortega-Sanchez IR, Bialek SR (2014). Congenital cytomegalovirus-related hospitalizations in infants < 1 year of age, United States, 1997–2009. Pediatr Infect Dis J..

[30] HCUP Databases. Healthcare Cost and Utilization Project. KID overview. 2018. https://www.hcup-us.ahrq.gov/kidoverview.jsp. Accessed 29 Oct 2018.

[31] Bureau of Labor Statistics (BLS). Databases, tables & calculators by subject. https://www.bls.gov/data. Accessed 4 Mar 2019.

[32] Dollard SC, Grosse SD, Ross DS (2007). New estimates of the prevalence of neurological and sensory sequelae and mortality associated with congenital cytomegalovirus infection. Rev Med Virol.

[33] Gold MR, Siegel JA, Russell LB, Weinstein, editors. Cost-effectiveness in health and medicine. New York: Oxford University Press; 1996.

[34] Luck SE, Wieringa JW, Blázquez-Gamero D, Henneke P, Schuster K, Butler K (2017). ESPID Congenital CMV Group Meeting, Leipzig 2015. Congenital cytomegalovirus: a European expert consensus statement on diagnosis and management. Pediatr Infect Dis J..

[35] Sweeten TL, Posey DJ, McDougle CJ (2004). Brief report: autistic disorder in three children with cytomegalovirus infection. J Autism Dev Dis..

[36] Williamson WD, Desmond MM, LaFevers N, Taber LH, Catlin FI, Weaver TG (1982). Symptomatic congenital cytomegalovirus: disorders of language, learning, and hearing. Am J Dis Child.

[37] Lanzieri TM, Leung J, Caviness AC, Chung W, Flores M, Blum P (2017). Long-term outcomes of children with symptomatic congenital cytomegalovirus disease. J Perinatol.

[38] Li R, Simmons KB, Bertolli J, Rivera-Garcia B, Cox S, Romero L (2017). Cost-effectiveness of increasing access to contraception during the Zika virus outbreak, Puerto Rico, 2016. Emerg Infect Dis.

[39] Popova S, Stade B, Bekmuradov D, Lange S, Rehm J (2011). What do we know about the economic impact of fetal alcohol spectrum disorder? A systematic literature review. Alcohol..

[40] Lavelle TA, Weinstein MC, Newhouse JP, Munir K, Kuhlthau KA, Prosser LA (2014). Economic burden of childhood autism spectrum disorders. Pediatrics.

[41] Wittenborn JS, Rein DB. Cost of vision problems: the economic burden of vision loss and eye disorders in the United States. 2013. https://www.preventblindness.org/sites/default/files/national/documents/Economic%20Burden%20of%20Vision%20Final%20Report_130611_0.pdf. Accessed 29 Oct 2018.

[42] Wang JT, Wang AY, Psarros C, Da Cruz M (2014). Rates of revision and device failure in cochlear implant surgery: a 30-year experience. Laryngoscope..

[43] Kancherla V, Amendah DD, Grosse SD, Yeargin-Allsopp M, Van Naarden Braun K (2012). Medical expenditures attributable to cerebral palsy and intellectual disability among Medicaid-enrolled children. Res Dev Disabil.

[44] Alarcon A, Martinez-Biarge M, Cabañas F, Hernanz A, Quero J, Garcia-Alix A (2013). Clinical, biochemical, and neuroimaging findings predict long-term neurodevelopmental outcome in symptomatic congenital cytomegalovirus infection. J Pediatr.

[45] Cameron NA, Gormley ME, Deshpande S (2013). Severity of disability in patients with cerebral palsy secondary to symptomatic congenital cytomegalovirus encephalopathy. J Pediatr Rehabil Med..

[46] Korndewal MJ, Oudesluys-Murphy AM, Kroes A, van der Sande MAB, de Melker HE, Vossen ACTM (2017). Long-term impairment attributable to congenital cytomegalovirus infection: a retrospective cohort study. Dev Med Child Neurol.

[47] Mitra S, Findley PA, Sambamoorthi U (2009). Health care expenditures of living with a disability: total expenditures, out-of-pocket expenses, and burden, 1996 to 2004. Arch Phys Med Rehabil.

[48] Cunningham PJ, Kohn L (2000). Health plan switching: choice or circumstance?. Health Aff.

[49] Baylor College of Medicine. Congenital CMV disease research clinic & registry. https://www.bcm.edu/departments/pediatrics/sections-divisions-centers/cmvregistry/. Accessed 14 Jan 2019.

