# Supplementary material for: A framework for assessing the lifetime economic burden of congenital cytomegalovirus in the United States
Source: Cost Eff Resour Alloc. 2019 Oct 3;17:21. doi: 10.1186/s12962-019-0189-0 (PMC6775673; doi:10.1186/s12962-019-0189-0)
Supplement: Supplementary file 1 — Additional file 1: Table S1. Gaps in cost components for estimating the economic burden of cCMVi. [file 12962_2019_189_MOESM1_ESM.docx]

Additional file 1: Table S1. Gaps in Cost Components for Estimating the Economic Burden of cCMVi

|  | Direct Costs | Indirect and Intangible Costs |
| --- | --- | --- |
| Birth/infancy | No non–health care costs included in cCMVi cost studies No studies found on influence of diagnosis on downstream costs | None found |
| Severe impairment with permanent disability | No costs found for patients with cCMVi  Cost estimates for proxy conditions are > 25 years old | None found |
| Mild to moderate impairment with development delay/disability | No costs found for patients with cCMVi | None found |
| Mild to moderate impairment with no development delay/disability | No costs found for patients with cCMVi | None found |
| Permanent disability | No costs found for patients with cCMVi | None found |

cCMVi = congenital cytomegalovirus infection.
